# Extensive retroperitoneal multiloculated abscesses after open cholecystectomy associated with bile leak: a case report

**DOI:** 10.1259/bjrcr.20210147

**Published:** 2022-04-29

**Authors:** Yuting Huang, Zarah Haleem, Harsh Patel, Ankur Pandey, Preetinder Singh Sandhu, Raymond Kim

**Affiliations:** 1 University of Maryland Medical Center Midtown Campus, Baltimore, Maryland, United States; 2 American University of Antigua College of Medicine, Coolidge, Antigua and Barbuda; 3 University of Maryland Medical Center, Baltimore, Maryland, United States

## Abstract

Retroperitoneal abscesses are rare and life-threatening. The incidence of abscesses after open cholecystectomies is <1% disregarding location. 11 cases reporting post-cholecystectomy retroperitoneal abscesses were archived on PubMed, 7 associated with gallstone retaining or spillage. Hereby, we present a case of extensive retroperitoneal multiloculated abscesses after open cholecystectomy complicated with bile leak, while no gallstone was noted. Early evaluation for retroperitoneal abscesses is critical if the patient does not clinically improve after cholecystectomy. Early treatment with drainage of the abscesses, antibiotics, and endoscopic retrograde cholangiopancreatography intervention to achieve source control can greatly improve the clinical outcome.

## Introduction

Retroperitoneal abscesses are rare but life-threatening. Symptoms are greatly variable and insidious which often lead to delayed diagnosis resulting in high morbidity and mortality.^1^ It is often caused by genitourinary infection or gastrointestinal (GI) causes, including inflammatory bowel disease, GI tract perforation and inflammation.^2^ Less often, they can develop following spinal osteomyelitis,^3^ necrotizing soft tissue infection,^4^ surgery and trauma.^5^ It is an uncommon complication of biliary tract procedures.^6^ Symptoms include abdominal/flank pain, decreased appetite, fever and chills, malaise and weight loss. Patients often have leukocytosis, elevated inflammatory markers and creatinine. Early drainage and eliminating the primary infectious source are crucial. We present a case with a worsening clinical course following an open cholecystectomy despite receiving broad-spectrum antibiotic treatment. The extensive retroperitoneal multiloculated abscesses likely extended from a bile leak. Urgent Interventional Radiology (IR)-guided abscess drainage and common bile duct (CBD) stenting via endoscopic retrograde cholangiopancreatography (ERCP) was performed to resolve the bile leak.

## Clinical presentation

**Figure 1.**
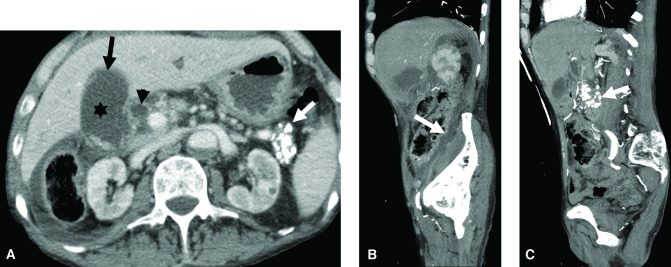
Abdominal CT pre cholecystectomy showing gangrenous cholecystitis. Axial (**A**) and sagittal (**B, C**) images demonstrating changes of acute gangrenous cholecystitis. There is a distended gallbladder with layering stones (asterisk, **A**), mural discontinuity in the fundal region (black arrow, **A**), and small pericholecystic fluid. There is a small amount of free fluid (arrow, **B**) extending from the right upper quadrant to the right lower quadrant without evidence of retroperitoneal abscess. Also note the intra- and extrahepatic (arrowhead, **A**) biliary dilation, and extensive pancreatic calcifications consistent with chronic pancreatitis (white arrow, **A, C**).

A 64-year-old African American male presented with 4 days of worsening abdominal pain, poor appetite, and shortness of breath. Past medical history included hypertension, opioid and alcohol use, and untreated hepatitis B and C, no history of pancreatitis. Physical exam was positive for hypotension and tachycardia. The patient was awake and oriented, however, ill-appearing, cachectic and incomprehensible, with mumbled monosyllabic responses. He has a body mass index (BMI) of 13, not on immunosuppressants, and HIV negative. Labs were remarkable for hemoglobin of 4.6 g dl^−1^, leukocytosis of 21,600/mL, thrombocytopenia of 78,000/mL, hyponatremia of 128 mmol l^−1^, hypokalemia of 3.1 mmol l^−1^, hyperuricemia of 105 mg dl^−1^, creatinine elevated to 1.91 mg dl^−1^, and lactic acidosis. AST 48 U l^−1^, ALT 14 U l^−1^, ALP 81 U l^−1^, Total bilirubin 0.9 mg dl^−1^. Prothrombin time, partial thromboplastin time, and international normalized ratio were within the normal range. Fecal occult blood test was positive. Pre-op CT-angiography abdomen/pelvis revealed chronic pancreatitis with extensive calcifications throughout the pancreas with a 0.7 × 1.5 cm cystic lesion in the pancreatic neck, distended gallbladder with a focal defect in the wall, intrahepatic and extrahepatic biliary dilation with CBD measured 1.2 cm, and free fluid around the gallbladder extending from the right upper quadrant to the right lower quadrant consistent with gangrenous cholecystitis. There was no evidence of retroperitoneal abscess pre-op (Figure 1). After platelet transfusion, urgent diagnostic open laparotomy was performed. Yellowish areas in the gallbladder wall indicate necrosis which has leaked. Duodenum and gallbladder hilum was covered with a lot of exudates up to the cystic duct, consistent with gangrenous cholecystitis. Cholecystectomy was performed, no damage or injury on the cystic duct or CBD during the procedure, and the surgical area was thoroughly washed before closing. Post-operatively, he received piperacillin-tazobactam, micafungin, and gentamicin. The patient’s leukocytosis and hypotension improved. He was successfully extubated on the third post-operative day, and transferred from the ICU to the medical floor.

**Figure 2.**
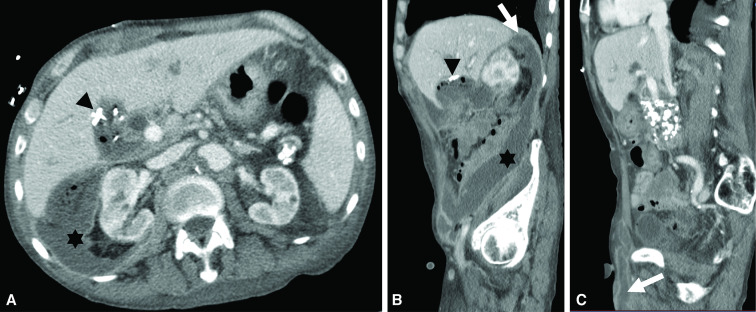
Abdominal CT post-cholecystectomy Day 8 showing extensive retroperitoneal multiloculated abscesses. Axial (**A**) and sagittal (**B, C**) images post-cholecystectomy with increasing leukocytosis. There is now extensive multiloculated right retroperitoneal abscess (asterisk, **B**) extending from the posterior liver margin at the level of the diaphragm (arrow, **B**) into the right lower quadrant anterior abdominal wall and scrotum (partially visualized, arrow, **C**) with contiguity with the gallbladder fossa that also contains fluid and gas along with surgical clips (arrowhead, **A, B**).

**Figure 3.**
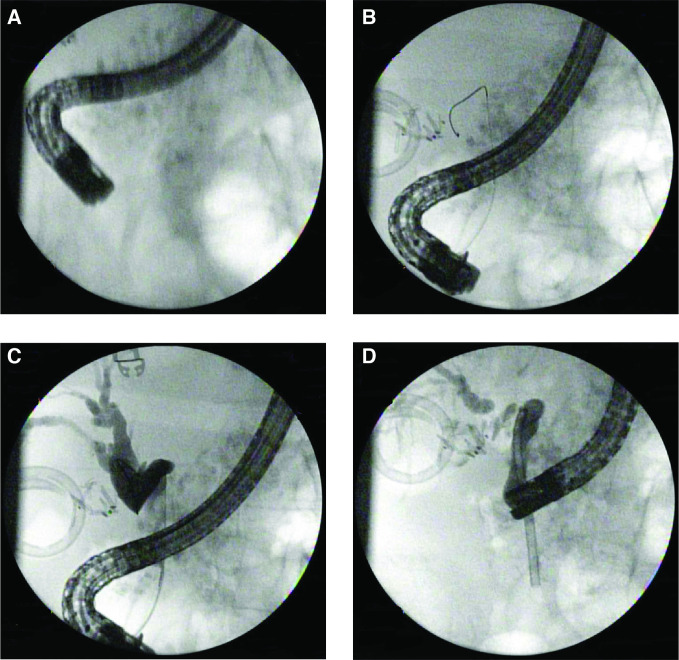
ERCP CBD stent placement to release the obstruction in the lower third CBD. (**A**) Scout film before cannulation, (**B**) bile duct cannulation with wire in CBD, (**C**) contrast injection to CBD-cholangiogram, biliary tract obstruction due to a kink in the lower third of the CBD. The upper third of the CBD and the common hepatic duct were dilated. (**D**) Bile duct stent placed, kink was resolved. CBD, common bile duct; ERCP, endoscopic retrograde cholangiopancreatography

He then had worsening abdominal pain, poor appetite, and drainage from the post-cholecystectomy abdominal drain site with episodic diarrhea. Ascitic cultures grew pan-sensitive *Klebsiella pneumoniae* and *Escherichia coli* requiring broad-spectrum antibiotics. The patient had deteriorating mental status. Abdominal CT scan on post-op Day 8 revealed extensive retroperitoneal multiloculated abscesses spanning from the diaphragm down to the upper scrotum (Figure 2). IR urgently placed JP drains. However, purulent drainage persisted. Hepatobiliary iminodiacetic acid (HIDA) scan revealed bile leak. Subsequent ERCP showed distal CBD obstruction but no overt bile leak and sphincterotomy was performed and one stent was placed into the CBD (Figure 3). Post-ERCP, the patient improved on intravenous antibiotics and a high caloric diet. Upon discharge, the patient continued ceftriaxone for 2 weeks and metronidazole for a total 4-week course with an infectious disease outpatient follow-up and ERCP 2 months later for stent removal.

## Discussion

Mortality rates associated with retroperitoneal infections remain high despite appropriate interventions.^7–9^ Risk factors include advanced age, male, malnutrition, obesity, diabetes, immunosuppressive status and malignancy. Abscesses formed in the retroperitoneum often spreads easily due to anatomically poor blood flow and communicating spaces.^6^ The lower-2/3 of CBD is located in the retroperitoneal space, therefore an impacted gallstone leads to CBD necrosis and perforation, or mechanical damage during cholecystectomy and/or CBD exploration increase the risk of bile leak resulting in bilious retroperitoneal infection.^10^ Abdominal CT remains the gold-standard in the diagnosis and detection of retroperitoneal abscesses, while CT combined with MRI can achieve sensitivity up to 100%.^11^ There is no guideline and consensus on the diagnostic criteria for retroperitoneal infections.^2^ Early infection source control, antibiotic treatment, and nutritional support are key to better outcomes.

Our case had risk factors including male, advanced age, and severe malnutrition. He presented with septic shock diagnosed with gangrenous cholecystitis. Gallbladder leak and pus-like exudate were observed during surgery, washout was performed thoroughly. The patient initially improved clinically on broad-spectrum antibiotics, however, he deteriorated again with worsening abdominal abscess likely due to ongoing bile leak. Ascitic culture was positive for commonly seen bacteria in biliary tract perforation and injury. Of note, majority of retroperitoneal abscess cases reported were associated with gallstone retaining or spillage, which was not seen in our case. The patient had benign dilation of CBD measured 1.2 cm before the surgery, with normal liver function panel throughout. We did not attempt to address the CBD dilation before the surgery due to patient had normal liver function panel, indicating no cholestatic damage was present, also the patient was critically ill with hypotension and tachycardia on admission. The fluid collection in right parabolic gutter in the pre-operative CT is at the same location as the abscess, bringing up the possibility that this could have been due to the pre-operative perforated gallbladder. The same fluid collection worsened after surgery in the setting of distal CBD stricture and continuously bile leak and led to the extensive retroperitoneal abscess formation. Even IR-guided JP drain was placed, the origin of the leak was not fully addressed until sphincterotomy and CBD stent placement via ERCP, that the drainage of bile was redirected to duodenum, and source control was eventually achieved. Nutritional support is important in patients with severe malnutrition,^12^ so a high caloric diet was given via PEG tube.

Another possible treatment route for this patient is percutaneous cholecystostomy. On admission the patient was critically ill, with abdominal CT showed a focal defect in the wall, and free fluid around the gall bladder, which is concerning for perforation. Complicated acute cholecystitis, including gallbladder gangrene/necrosis, perforation, and emphysematous cholecystitis, may be fatal, and is indications for emergency cholecystectomy. Recently, Dr Wadhwa et al reported the use of percutaneous cholecystostomy is increasing among patients admitted with acute cholecystitis.^13^ However, in the randomized CHOCOLATE trial, the investigators compared laparoscopic cholecystectomy with percutaneous cholecystostomy for high-risk acute cholecystitis and found a higher risk of complications, higher reintervention rate, and higher rate of recurrent biliary disease in the percutaneous drainage group.^14^

In conclusion, retroperitoneal abscess is a rare and dangerous complication after cholecystectomies due to their insidious onset and location, typically due to a perforated gallbladder and spilled stones. In our case, the patient kept on deteriorating even with appropriate broad-spectrum antibiotics treatment and timely IR-guided gallbladder drainage, indicating insufficient source control. Early repeat image study is essential in diagnosis of retroperitoneal abscess. Treatments, including endoscopic methods, should be attempted to achieve prompt infection source control, which can be life-saving in persistent retroperitoneal infection.

## Learning points

- Retroperitoneal abscess is a rare and life-threatening complication after cholecystectomies.
- Early diagnosis with abdominal CT and prompt infection source control is life-saving in persistent retroperitoneal infection.
- IR-guided drainage and endoscopic approach are often used in source control.
